# Pharmacological Characteristics and Clinical Applications of K201

**DOI:** 10.2174/157488409788184972

**Published:** 2009-05

**Authors:** Noboru Kaneko, Ryuko Matsuda, Yoshihito Hata, Ken Shimamoto

**Affiliations:** 1 Department of Cardiology and Pneumology, Dokkyo Medical University, Tochigi, Japan; 2 Department of Cardiology, Tokyo Women’s Medical University, Aoyama Hospital, Tokyo, Japan

**Keywords:** K201, cardiac ryanodine receptor (RyR2), annexin V, diastolic heart failure, multiple-channel blocker.

## Abstract

K201 is a 1,4-benzothiazepine derivative that is a promising new drug with a strong cardioprotective effect. We initially discovered K201 as an effective suppressant of sudden cardiac cell death due to calcium overload. K201 is a nonspecific blocker of sodium, potassium and calcium channels, and its cardioprotective effect is more marked than those of nicorandil, prazosine, propranolol, verapamil and diltiazem. Recently, K201 has also been shown to have activities indicated for treatment of atrial fibrillation, ventricular fibrillation, heart failure and ischemic heart disease, including action as a multiple-channel blocker, inhibition of diastolic Ca^2+^ release from the sarcoplasmic reticulum, suppression of spontaneous Ca^2+^ sparks and Ca^2+^ waves, blockage of annexin V and provision of myocardial protection, and improvement of norepinephrine-induced diastolic dysfunction. Here, we describe the pharmacological characteristics and clinical applications of K201.

## INTRODUCTION

Ischemic heart disease, heart failure and severe arrhythmia still have a poor outcome despite recent advances in drug therapy. These diseases interact with one another and consequently treatment options are often limited. In two large-scale clinical trials, the CAST [1] and SWORD [2] studies, performed to assess the effect of antiarrhythmic drugs on myocardial infarction, Vaughan-Williams Class Ic and III drugs aggravated outcome more severely than placebo and limitations of specific single-channel blockers were found.

K201 [4-[3-(4-benzylpiperidin-1-yl)propionyl]-7-methoxy-2,3,4,5-tetrahydro-1,4-benzothiazepine monohydrochloride; C_25_H_32_N_2_O_2_S•HCl; MW: 461.07 (Fig. **l**)] is a promising new drug that has cardioprotective effects. K201 was initially discovered by Kaneko et al. as an effective suppressant of sudden cardiac cell death (that is, kinetic cell death) for prevention of onset of myocardial infarction [3]. Kinetic cell death occurs due to myofibrillar overcontraction induced by high intracellular calcium, but not ischemic reperfusion. We first established a screening system for drug discovery using a kinetic cell death model prepared by the Langendorff method. In this model, myofibrillar overcontraction due to excessive Ca^2+^ release from the sarcoplasmic reticulum was induced by stimulation of epinephrine and caffeine with Ca^2+^ loading. Many compounds were screened using this model, with the resultant discovery of K201 [3]. 

K201 is a 1,4-benzothiazepine derivative with a different structural scaffold from the 1,5-benzothiazepine, diltiazem, and is not classified as a calcium channel blocker. Rather than acting as a β receptor blocker, K201 blocks α_1_ receptors and intracellular Ca^2+^ pathways [3] and has various actions.

The effects of K201 on the cardiovascular system have been studied extensively and the sites of action of K201 in membranes and intracellular organs in cardiomyocytes are shown in Fig. (**2**). We note that the drug is currently referred to as K201 or JTV519. K201 was the original name of the compound during development, whereas the drug was referred to as JTV519 in later clinical trials. Here, we describe the pharmacological characteristics and clinical applications of K201. 

## PHARMACOLOGICAL CHARACTERISTICS OF K201

### 1. K201 is a Multiple-Channel Blocker

K201 is a non-specific blocker of sodium, potassium and calcium channels [4-6]. The effect of K201 on membrane current has been studied using the whole-cell voltage-clamp method in isolated guinea pig ventricular myocytes [4, 5] and in atrial cells [6]. K201 inhibits the Na^+^ current (I_Na_) in a voltage- and frequency-dependent manner with IC_50_ values of approximately 1.2 and 2 μM at holding potentials of -60 and -90 mV, respectively. The time-course of I_Na_ blocking by K201 is slower than that of lidocaine and similar to that of quinidine; therefore, K201 is categorized as having “intermediate” but not “fast” kinetics.

K201 also slightly blocks the Ca^2+^ current (I_Ca_) and the inwardly rectifying K^+^ current (I_K1_) with IC_50_ values of 3 and 5 μM, respectively. K201 blocks the rapidly activating component of the delayed rectified K^+^ current (I_Kr_) with an IC_50_ of 1.2 μM [6], but not the slowly activating component (I_Ks_) [4-6]. Recently, Nakaya et al. [6] also showed inhibitory effects of K201 on the muscarinic acetylcholine receptor-operated K current (I_KAch_), and K201 inhibits the carbachol-, adenosine-, and GTP gamma S-induced I_KAch _with IC_50_ values of 0.12, 2.29 and 2.42 μM, respectively [6]. 

### 2. K201 Inhibits Ca^2+^ Release from the Sarcoplasmic Reticulum

Calcium release from the sarcoplasmic reticulum (SR) is the major source of Ca^2+^ required for excitation-contraction (EC) coupling. Membrane depolarization activates the L-type Ca^2+^ influx channel, which results in Ca^2+ ^influx that then triggers a large Ca^2+^ release from the SR by opening the cardiac ryanodine receptor (RyR2). This mechanism is known as Ca^2+^-induced Ca^2+^ release [7]. 

 K201 is believed to stabilize the closed state of RyR2 by increasing its affinity for the 12.6 kDa FK506-binding protein (FKBP12.6), which prevents the Ca^2+^ leak that triggers arrhythmias [8]. Protein kinase A (PKA) phosphorylation of RyR2 dissociates FKBP12.6 and regulates channel opening. In failing hearts, RyR2 is hyperphosphorylated by PKA, resulting in defective channel function due to increased sensitivity to Ca^2+^-induced activation [8-10]. Hyperphosphorylation of RyR2 in heart failure produced by rapid right ventricular pacing is reversed by K201 treatment, with a return to levels of channel phosphorylation seen in normal hearts. K201 also prevents a decrease in the amount of RyR2-bound FKBP12.6 in this model [11]. However, recent studies have shown that FKBP12.6 may not be involved in Ca^2+^ release from the sarcoplasmic reticulum, since the loss of FKBP12.6 has no significant effect on the conduction and activation of RyR2 or the propensity for spontaneous Ca^2+^ release and stress-induced ventricular arrhythmias [12]. K201 corrects the defective channel gating in RyR2 and causes a rapid conformational change and increased Ca^2+^ release in heart failure [13]. However, it is not clear if this mechanism is associated with FKBP12.6 in terms of channel stabilization of RyR2 [13]. In a Ca^2+^ overload rat model, treatment of ventricular myocytes with FK506 to dissociate FKBP12.6 from RyR2 did not affect the suppression of spontaneous Ca^2+^ release by K201 [14].

The phosphorylation status of RyR2 in heart failure and its effect on the RyR2-FKBP12.6 interaction are controversial. The PKA-dependent increase in myocyte Ca^2+^ spark frequency and size is entirely attributable to phospholamban phosphorylation and consequent enhanced SR Ca^2+^ loading, and PKA does not seem to have any appreciable effects on resting RyR2 function in ventricular myocytes [15]. In cardiac SR vesicles, K201 restores the conformational change and corrects defective channel gating in RyR2 independent of phosphorylation of RyR2 [13]. FKBP12.6 dissociates from RyR2 due to PKA phosphorylation or FK506-induced domain unzipping, and this causes Ca^2+^ leak. K201 reverses this domain unzipping and stops Ca^2+^ leak completely, even after FKBP12.6 has mainly dissociated from RyR2 due to hyperphosphorylation of RyR2 [16]. 

### 3. K201 Suppresses Spontaneous Ca^2+^ Sparks (Ca^2+^ leak) and Ca^2+^ Waves

In heart muscle, local elevations in intracellular calcium (Ca^2+^ sparks) are elementary SR Ca^2+^-release events. A Ca^2+ ^spark is a local Ca^2+ ^release from the SR that also occurs in normal ventricular myocytes. Ca^2+ ^spark accumulation propagates to an adjacent SR as a Ca^2+^ wave, with consequent induction of Ca^2+^-induced Ca^2+^ release [17, 18]. In the 48-hour infarcted canine heart, abnormal Ca^2+^ wave activity is the underlying cause of afterdepolarization-induced electrical activity in surviving subendocardial Purkinje cells. Drugs that block inward sodium or calcium currents (verapamil, tetrodotoxin) have no effect on Ca^2+^ activity in Purkinje cells, but K201 strongly suppresses spontaneous Ca^2+ ^release in Purkinje cells dispersed from the subendcardium of the infarct zone (IZPCs) [19]. Agents that block or inhibit intracellular Ca^2+^ channel activity and reduce Ca^2+^ waves can have antiarrhythmic effects in arrhythmogenic Purkinje cells in the infarcted heart [19]. K201 at 1 μmol/L reduces the frequency of spontaneous Ca^2+^ release and the frequency and velocity of SR Ca^2+^ waves, despite the absence of a change in SR Ca^2+^ content, and also reduces Ca^2+^ spark amplitude and frequency and inhibits SR Ca^2+^ uptake and release. K201 inhibits spontaneous diastolic Ca^2+^ release during Ca^2+^ overload by a dual inhibitory action on sarcoplasmic reticulum Ca^2+^-ATPase (SERCA2a) and RyR2, without significantly affecting the transient Ca^2+^ amplitude [20]. 

### 4. K201 Blocks Annexin V and Provides Myocardial Protection

**Fig. (3):**
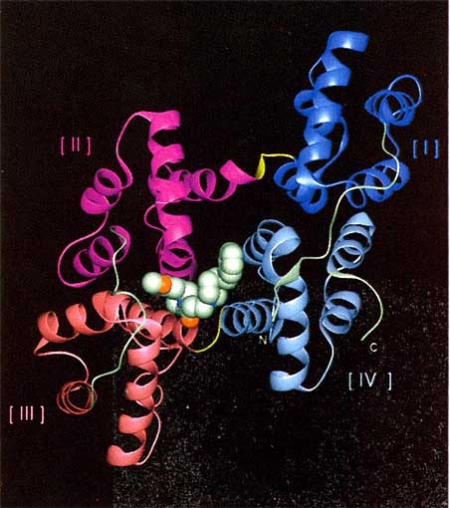
The crystal structure of annexin V and binding site of K201. The K201 molecule is shown as a space-filling model. Hinge regions are represented in yellow and domains I, II, II and IV are colored dark blue, magenta, orange and sky-blue, respectively [24].

**Fig. (4):**
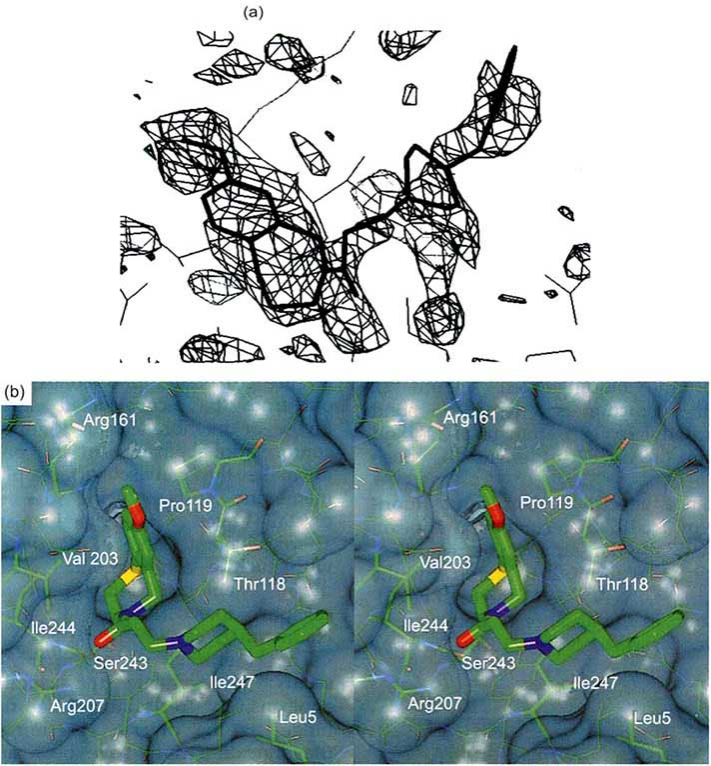
Amino acids of annexin V in the vicinity of bound K201. (**a**) The L-shaped K201 molecule interacts at the hinge 2 region. (**b**) The K201 binding site in stereo view [24].

Annexin V is a member of the annexin family of Ca^2+^-binding proteins that have activity that depends on the Ca^2+^ concentration. *In vitro*, annexin V binding to F-actin depends on the calcium concentration and the presence of phosphatidylserine, a membrane phospholipid [21-23]. K201 inhibits the interaction between these two proteins, whereas diltiazem and KT362 do not inhibit this binding [3]. Annexin V is distributed in various organs and cells and is involved in processes including fusion, development and differentiation. This protein induces calcium channel activity, as shown by patch-clamping, and is critical in cellular signaling. The crystal structure of annexin V bound to K201 shows that the drug binds in the Ca^2+^ “hole” of annexin V (Fig. **3**) [24]. K201 binds in an L-shaped conformation at the hinge region cavity formed by the N-terminal strand and domains II, III and IV, on the side opposite to the calcium and membrane-binding surface (Fig. **4**(**a**),(**b**)). K201 may restrain the hinge movement of annexin V in an allosteric manner, resulting in inhibition of calcium movement across the annexin V molecule [24]. We have shown that annexin V activates Ca^2+^ channels and K201 inhibits this Ca^2+^ influx in an artificial lipid membrane. Annexin V also induces Ca^2+^ channel activity in large unilamellar vesicles (LUVs) and K201 shows dose-dependent inhibition of annexin V-induced inward Ca^2+^ movement into LUVs [25].

A recent study has shown a similarity of the amino-acid sequences of RyR2 and annexin V: the K201 binding site in annexin is present as domain 2114-2149 of RyR2, and domain 2059-2156 of RyR2 showed a striking similarity to the corresponding sequence of annexin V [26]. Interruption of the interdomain interaction between domain 2114-2149 and the central domain 2234-2750 seems to mediate stabilization of RyR2 in failing hearts, and K201 inhibition of Ca^2+^ leakage does not depend on the facilitated binding of FKBP12.6 to RyR2 [26]. Another recent study has shown that annexin V has a site homologous to the δ protein kinase C (PKC)-binding protein RACK (receptor for activated C kinase). The RACK-like sequence in annexin V is in close proximity to the K201 binding site on annexin V, which indicates the potential importance of the RACK-like sequence in annexin V for PKC binding. δPKC binding to annexin V activates δPKC translocation, and therefore K201 may inhibit δPKC activity [27].

### 5. K201 Improves Norepinephrine-Induced Diastolic Dysfunction

**Fig. (5):**
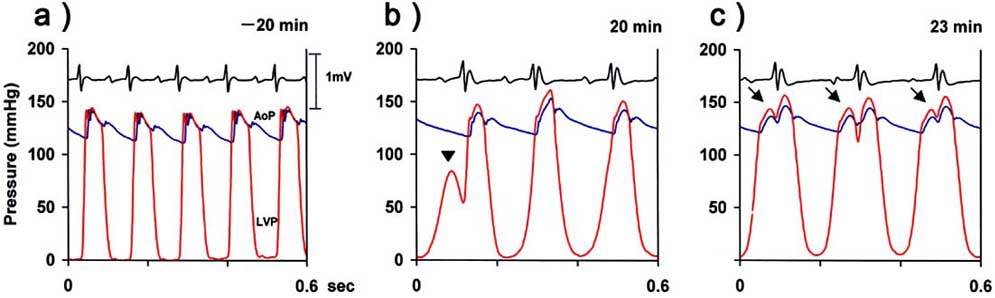
Simultaneous recording of ventricular and aortic pressure in rats. **A**) Before Ca^2+^ loading, **B**) 20 minutes after norepinephrine administration with Ca^2+^ loading, and **C**) 23 minutes after norepinephrine administration with Ca^2+^ loading [28]. Diastolic contracture (B, arrow) was observed after norepinephrine administration with Ca^2+^ loading, and subsequently the diastolic pressure exceeded the aortic pressure, resulting in opening of the aortic valve in the diastolic phase (C, arrow) [28]. K201 inhibits this diastolic contracture, but diltiazem does not do so [29]. The figure is modified from that shown in ref. 28.

We have shown that norepinephrine with Ca^2+^ loading induces severe diastolic dysfunction [28]. Under these conditions, a diastolic pressure equivalent to the systolic pressure is induced without membrane depolarization. The left ventricular diastolic pressure is markedly increased and the diastolic pressure exceeds the aortic pressure, resulting in opening of the aortic valve in the diastolic phase (Fig. **5**) [28]. K201 significantly inhibits norepinephrine-induced diastolic dysfunction whereas diltiazem has no suppressive effect [29]. Norepinephrine administration in old myocardial infarction rats induces similar diastolic dysfunction. An intracellular Ca^2+^ increase in the diastolic phase is an important factor in development of diastolic dysfunction, but it has also been reported that an intracellular Ca^2+^ increase in the diastolic phase alone does not increase the left ventricular end-diastolic pressure [30].

## CLINICAL APPLICATIONS OF K201

### 1. Inhibition of Atrial Fibrillation

K201 inhibits carbachol-induced atrial fibrillation in guinea pig atrial muscle [6]. An increase in I_KAch_ and I_Kr_ in atrial muscle cells is a potential mechanism for atrial fibrillation, and I_KAch_ is often generated in the sinus node and atrium, and particularly in the atrial appendage of the atrium. K201 directly inhibits I_KAch_ [6] and also inhibits the atrial I_Kr_ with an IC_50_ of 0.41 μM. Therefore, K201 is thought to improve atrial fibrillation by inhibiting I_KAch_ and I_Kr_ [6], and has been shown to inhibit experimental atrial fibrillation in a dog aseptic pericarditis model [31].

Abnormal calcium homeostasis with ryanodine receptor dysfunction may underlie arrhythmogenic activity in pulmonary veins. Both pretreatment and rapid administration of K201 (0.3 μM) decrease isoproterenol-induced arrhythmogenesis in pulmonary veins through reduction of the arrhythmogenic activity of pulmonary vein cardiomyocytes. These findings indicate the anti-arrhythmic potential of K201 [32].

### 2. Inhibition of Ventricular Tachycardia and Fibrillation

Triggered activity is a major cause of arrhythmias, in addition to abnormal automaticity and reentry. Triggered activity is classified into early after depolarization (EAD), a transient depolarization that is generated immediately after or from the second or third phase of the preceding action potential; and delayed after depolarization (DAD) generated from the fourth phase of the preceding action potential. EAD develops due to a prolonged duration of the action potential and DAD is induced by an abnormal increase in intracellular Ca^2+^. Classic antiarrhythmic drugs tend to inhibit cardiac function and induce arrhythmia. The class III drug clofilium prolongs QT and induces torsades de pointes (Tdp) and ventricular tachycardia when combined with an α_1_-adrenoceptor stimulator, due to EAD generated from the third phase of the action potential [33-35]. We have shown that K201 inhibits clofilium-induced Tdp and attenuates the prolongation of repolarization caused by clofilium. Our results show that K201 causes prolongation of the QT and QTc intervals, but does not induce Tdp, in the presence or absence of α_1_-adrenoceptor stimulation. The inhibition of clofilium-induced Tdp by K201, despite QT prolongation, suggests that QT prolongation alone is not a proarrhythmic signal. K201 suppresses Tdp dose-dependently by blocking the α_1_-adrenoceptor and inhibiting abnormal Ca^2+^ release from the SR [36].

### 3. Improvement of Diastolic Heart Failure

Diastolic dysfunction refers to an abnormality in diastolic distensibility, filling, and relaxation of the left ventricle [37, 38]. The onset mechanism of this type of heart failure, (so-called diastolic heart failure) is unknown. Systolic heart failure can be treated with digitalis and catecholamines that enhance myocardial contractility, but there are no drugs for treatment of diastolic heart failure. We have reported that norepinephrine induces a marked increase in left ventricular end-diastolic pressure in rats with Ca^2+^ loading or old myocardial infarction [28]. K201 significantly suppressed the increase in left ventricular end-diastolic pressure, decreased the incidence of norepinephrine-induced diastolic contracture, including aortic value opening in the diastolic phase, and improved Ea and deceleration time in cardiac Doppler ultrasonography. These results suggest that K201 may be an agent for treatment of diastolic heart failure [11, 29, 39].

### 4. Cardioprotective Effects Against Myocardial Injury

K201 was initially developed as a drug that prevents onset of myocardial infarction and sudden cardiac death. K201 provides myocardial protection more effectively than existing calcium channel blockers and β blockers and is less negatively inotropic and chronotropic [3]. K201 inhibits ischemia/reperfusion injury dose-dependently [40] and blocks (Takotsubo) stress cardiomyopathy induced by large amounts of isoproterenol (5 mg/kg) [41]. K201 also has a cardioprotective effect against myocardial injury that is stronger than those of nicorandil, prazosin, propanolol, verapamil and diltiazem [3]. These results indicate that K201 can prevent myocardial injury caused by ischemia and catecholamines.

## Figures and Tables

**Fig. (1):**
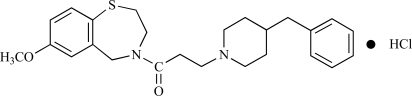
Molecular structure of K201.

**Fig. (2):**
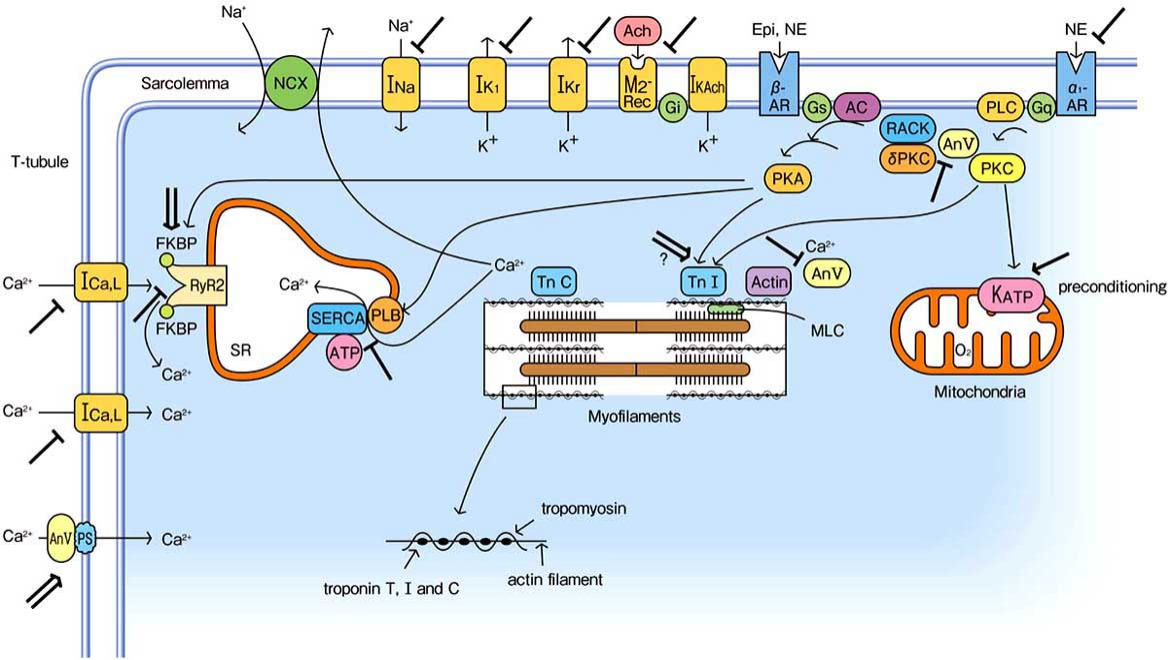
Action sites of K201 on the cell membrane and in cells: ⊥, inhibition; ↓, activation; ⇓, modulation. AC: adenylate cyclase, Ach: acetylcholine, An V: annexin V, AR: adrenoreceptor, Epi: epinephrine, FKBP: FK506 binding protein, G: G-protein, MLC: myosin light chain, M_2_-Rec: M_2_-muscarinic receptor, NCX: Na^+^/Ca^2+^ exchanger, PKA: protein kinase A, PKC: protein kinase C, PLB: phospholamban, PLC: phospholipase C, PS: phosphatidylserine, NE: norepinephrine, RACK: receptor for activated C kinase, RyR2: ryanodine receptor 2, SR: sarcoplasmic reticulum, SERCA: SR Ca^2+^-ATPase, Tn: troponin.
